# Genome Analysis of Rhodococcus Sp. DSSKP-R-001: A Highly Effective *β*-Estradiol-Degrading Bacterium

**DOI:** 10.1155/2018/3505428

**Published:** 2018-10-28

**Authors:** Hongyan Zhao, Kejian Tian, Qing Qiu, Yu Wang, Hongyan Zhang, Shuang Ma, Shenbao Jin, Hongliang Huo

**Affiliations:** ^1^School of Life Sciences, Northeast Normal University, No. 5268, Renmin Main Street, Nanguan District, Changchun City, Jilin Province, China; ^2^School of Environment, Northeast Normal University, No. 2555 Jingyue Avenue, Changchun City, Jilin Province, China

## Abstract

We screened bacteria that use E2 as its sole source of carbon and energy for growth and identified them as Rhodococcus, and we named them DSSKP-R-001. For a better understanding of the metabolic potential of the strain, whole genome sequencing of Rhodococcus DSSKP-R-001 and annotation of the functional genes were performed. The genomic sketches included a predicted protein-coding gene of approximately 5.4 Mbp with G + C content of 68.72% and 5180. The genome of Rhodococcus strain DSSKP-R-001 consists of three replicons: one chromosome and two plasmids of 5.2, 0.09, and 0.09, respectively. The results showed that there were ten steroid-degrading enzymes distributed in the whole genome of the strain. The existence and expression of estradiol-degrading enzymes were verified by PCR and RTPCR. Finally, comparative genomics was used to compare multiple strains of Rhodococcus. It was found that Rhodococcus DSSKP-R-001 had the highest similarity to Rhodococcus sp. P14 and there were 2070 core genes shared with Rhodococcus sp. P14, Rhodococcus jostii RHA1, Rhodococcus opacus B4, and Rhodococcus equi 103S, showing evolutionary homology. In summary, this study provides a comprehensive understanding of the role of Rhodococcus DSSKP-R-001 in estradiol-efficient degradation of these assays for Rhodococcus. DSSKP-R-001 in bioremediation and evolution within Rhodococcus has important meaning.

## 1. Introduction

Endocrine-disrupting effects of environmental chemistry have increasingly attracted the attention of the academic community [1, 2]. A large amount of survey data shows that there are many substances in the environment that can simulate and interfere with the secretion function of animals and humans [3, 4]. Once these exogenous compounds enter the body, they interfere with endocrine material synthesis, release, transport, metabolism, and binding processes and can activate or inhibit the function of the endocrine system, thus undermining the body's environmental stability. These compounds are called endocrine-disrupting chemicals [5]. Among the environmental endocrine disruptors, the chemicals that mimic estrogen the most are known as environmental estrogens, a large class of estrogen-like chemicals [6]. To date, more than 70 compounds have been classified as environmental estrogens [7]. 17*β*-Estradiol (E2), a natural estrogen, is also a typical environmental endocrine disrupter and is the strongest one of many environmental hormones. It is commonly found in various environments, especially in water [8]. E2 has irreversible effects on both humans and animals. In particular, fertilized eggs, immature individuals, and juveniles are more susceptible to the effects of E2. As E2 is mainly distributed in the aquatic environment, it is particularly harmful to aquatic organisms [9]. Some studies have shown that E2 has a serious impact on the growth and development of aquatic organisms. Even extremely low concentrations of E2 (ng/L) can cause females to become males [10, 11]. Biegel and colleagues found that adult mice had abnormal ovarian and testicular development in E2-contaminated environments [12]. Vajda et al. found abnormalities in the reproduction of fish living in wastewater containing E2 [11, 13]. In addition, the study also found that E2 pollution and female and male prostate cancer increased the incidence of testicular cancer, which has a direct link [14, 15]. It is therefore crucial to better find “destiny” in the environment and to develop effective governance approaches [16].

Rhodococcus has a wide range of metabolic capabilities, such as the degradation and transformation of various environmental pollutants [17, 18]. An important feature of Rhodococcus is the ability to degrade a range of naturally occurring steroid hormones, including cholesterol and phytosterols, such as beta-sitosterol [19, 20]. In recent years, more and more reports have been published about the degradation of estrogen by Rhodococcus [20, 21]. Key enzymes involved in estrogen degradation have also been reported [22]. Related reports show that estrogen degradation is achieved by the participation of multiple enzymes.

However, at present, the understanding of the related enzymes capable of degrading estrogen is limited to a relatively small number, and such a lack of research hinders the functional enzyme environment that helps to repair estrogen contamination. Our research team isolated and purified a strain of E2, which is the only carbon and energy source. The strain was identified as Rhodococcus by 16S rRNA and named Rhodococcus DSSKP-R-001 and is deposited with the China National Center for Microbiology as CGMCC No. 12392. This study focuses on the characterization and analysis of E2-degrading enzymes in Rhodococcus DSSKP-R-001. First, E2 was used as the sole carbon source and strain Rhodococcus DSSKP-R-001 was used to test the growth curve to determine the efficiency of its degradation. Next, the strains were sequenced for three generations to analyze the genome and annotate the functional genes to find E2-degrading enzymes and partition all the enzymes. Subsequently, PCR and RTPCR were used to validate the degradative enzymes. Finally, comparative genomics were used to characterize the evolutionary homology. This article is a study of E2-degrading enzymes involved in a large number of types of research and lay the foundation for the future degradation of estrogen.

## 2. Experimental Materials and Methods

### 2.1. Experimental Drugs and Reagents

The experiment drugs and reagents used in this word include 17*β*-estradiol (E2) (purity 98% produced by Shanghai Jingjing Biochemical Technology Co., Ltd.), methanol (Beijing Chemical Factory), and the bacterial genomic DNA extraction kit (Tiangen Biochemical Technology Co., Ltd.).

### 2.2. Experimental Methods

#### 2.2.1. Strain Selection

A sample of estrogen from the pharmaceutical factory in Beijing and 1 g of soil samples were added to a concentration of 5 mg/L of estradiol selection medium (10 g/L estradiol mother liquor was added to 100 mL of inorganic salt medium, and estrogen was used to prepare a final concentration of alcohol of 5 mg/L, 10 mg/L, 20 mg/L, 40 mg/L, 50 mg/L, 60 mg/L, 80 mg/L, and 100 mg/L. The medium was placed in a constant temperature water bath until the methanol was completely evaporated, leaving inorganic salt medium (g/L): 4.26 g Na_2_HPO_4_, 2.65 g KH_2_PO_4_, 0.2 g MgSO_4_ · 7H_2_O, and 1.5 g (NH4). One mL of trace elements was added and diluted to 1000 mL with distilled water). After shaking the culture at 30°C and 120 rpm for 72 h, 200 *μ*L of the culture medium was inoculated into 10 mg/L estradiol selective medium and cultured under the same conditions for 72 h. In this way, the culture was continued in medium containing 20 mg/L, 40 mg/L, 60 mg/L, 80 mg/L, and 100 mg/L estradiol. Using the dilution coating plate method, we diluted 1 mL of bacterial liquid at different concentrations of 10-2-10-8 with distilled water and applied the diluted bacterial liquid of 10-2-10-8 to the estradiol concentration of 100 mg/L LB solid medium (10 g tryptone, 5 g yeast extract, and 10 g NaCl, adjust pH to 7.4 with 0.1 mol/L NaOH and 0.1 mol/L HCl, dilute to 1000 mL with distilled water, and add 2% agar), which was placed in a thermostatic incubator at 30°C for 3 days. Colonies of different shapes and colors were picked and streaked on LB solid medium with an estradiol concentration of 100 mg/until a single colony is separated.

#### 2.2.2. Strain 16S rDNA Identification

The 16S rDNA sequence of the strain was PCR-amplified by polymerase chain reaction (PCR). The 16S rDNA universal PCR primer sequence is as follows: 5′ → 3′ upstream AGAGTTTGATCMTGGCTCAG and downstream TACGGYTACCTTGTTACGACTT.

#### 2.2.3. Strain Preservation

Then, 60% glycerol was added to the cryopreservation tube, autoclaved at 121°C for 30 min, and used as a protective solution. The ratio of bacterial liquid to glycerol was 1 : 1 (v/v), stored at −80°C, and deposited at the Beijing Institute of Microbiology.

#### 2.2.4. Determination of Estrogen Concentrations

Estradiol standard curve preparation used concentrations of 10 mg/L, 20 mg/L, 40 mg/L, 60 mg/L, and 80 mg/L of estradiol. High-performance liquid chromatography was used to measure the content and draw the standard curve. Preparation of the bacterial suspension involved the strain Rhodococcus DSSKP-R-001 inoculated on LB liquid medium at 30°C, 120 rpm for 24 h. Then, the bacterial liquid was centrifuged at 4000 rpm for 10 min, the supernatant was discarded, the bacterial cell pellet was washed with 0.2 M phosphate buffer (pH 7.0), the sample was centrifuged at 4000 rpm for 10 min, and the supernatant was discarded. After repeated washing three times, 0.2 M phosphate-buffered solution (pH 7.0) was finally used to dilute the bacterial liquid to prepare a bacterial suspension with an OD600 nm value of 1.0 for further use. The bacterial suspension was added to a concentration of 50 mg/L estradiol selection medium at 3% (v/v) inoculum size and cultured at 30°C and 120 rpm for 6 days. Samples were taken every 24 h. After the samples were pretreated, the remaining concentration of estradiol in the medium was measured by high-performance liquid chromatography, and the cell density was measured at OD600 using a microplate reader.

#### 2.2.5. Genomic DNA Extraction

The strain was inoculated in LB liquid medium at 30°C, 120 rpm, after culturing for 24 h, and centrifuged at 4000 rpm for 10 min. The supernatant was discarded, the cells were resuspended with 0.2 M phosphate buffer (pH 7.0), the sample was centrifuged again, and the precipitate was collected. Genomic DNA was extracted by a bacterial genomic DNA extraction kit, and the DNA concentration was determined by NanoDrop ND-1000 ultramicro-UV spectrophotometer.

#### 2.2.6. Genome Sequencing and Assembly

The strain Rhodococcus DSSKP-R-001 was inoculated into LB liquid medium, incubated at 30 rpm and 120 rpm for 24 h, and centrifuged at 4000 rpm for 10 min; the supernatant was discarded; the cells were resuspended in 0.2 M phosphate buffer (pH 7.0); and the sample was centrifuged again. This process was repeated three times, and the sediment was collected. A bacterial genomic DNA extraction kit was used for the extraction of genomic DNA. The DNA concentration was determined using an ultra-trace UV spectrophotometer (NanoDrop ND-1000). The DNA sample of the Rhodococcus strain DSSKP-R-001, which was tested by electrophoresis, was cut by a Covaris g-TUBE into fragments of the desired size for construction of the library. After DNA damage repair and terminal repair, a hairpin-type linker was ligated using DNA ligase purification of the DNA fragments using AMPure PB beads at both ends of the DNA fragment to construct the SMRTbell library. The constructed library was quantified by Qubit, and the size of the inserted fragment was detected with Agilent 2100, followed by sequencing using the PacBio RSII platform. Whole genome sequencing was performed using single-molecule sequencing. The raw data obtained by sequencing was filtered to obtain valid data. Starting from the Clean Data with various quality controls, SMRT portal software was used to perform genome assembly on the reads to obtain the initial assembly results. The reads were aligned to the assembly sequence, and the distribution of the sequencing depth was calculated. Based on the sequence length and alignment method, we discriminated whether the initial assembled sequence is chromosomal or a plasmid sequence and tested whether the sequence is looped.

#### 2.2.7. Genome-Wide Gene Annotation

Gene islands were predicted with GeneMarkS software and IslandPath-DIOMB software, rRNAmmer software was used to predict rRNA, tRNAscan software was used to predict the tRNA region and tRNA secondary structure, Rfam software was used to predict sRNA, RepeatMasker software was used to predict scattered repeat sequences, and TRF software was used to predict tandem repeats. The amino acid sequence of the strain Rhodococcus DSSKP-R-001 was compared with the COG, GO, KEGG, NR, and Swiss-Prot databases using NCBI's BLAST software, and its genes and corresponding functional annotation information were combined to obtain the annotation result. The complete genome of the Rhodococcus DSSKP-R-001 strain was constructed using Circos software combined with the prediction of encoding gene, noncoding RNA, and gene function annotation.

#### 2.2.8. PCR and RTPCR

Amplification was performed using EasyTaq® DNA Polymerase (Cat. No.: AP111-03) and High Pure dNTPs (Cat. No.: AD101-02) from Beijing AllGen Biotech Co., Ltd. The primers are shown in Table 1. PCR amplification conditions were as follows: 94°C for 5 min, 94°C for 30 sec, 55°C for 30 sec, 72°C for 90 sec, and 72°C for 10 min for 35 cycles. The resulting PCR product was subjected to agarose gel electrophoresis. Afterwards, the corrected PCR product was recovered using a Gel Extraction Kit (Cat. No.: D2500-02) and a reagent from Omega Bio-tek Nucleic Acid Purification Company. Finally, the corresponding amplification primers were used for sequencing. Reverse transcriptase amplification of the E2-degrading enzyme gene strain DSSKP-R-001 was inoculated into an inorganic salt medium with E2 as the sole carbon source, where the E2 concentration was 50 mg/L, at 30°C and 120 rpm. The total RNA was extracted from the total RNA. The RNA was converted into cDNA using the RT-PCR kit, and the cDNA was used as a template for PCR. The PCR reaction primers are shown in Table 2 under the following reaction conditions: 35 cycles of 94°C for 30 s, annealing at 55°C for 30 s, and extension at 72°C for 90 s, with a final extension at 72°C for 10 min and holding at 4°C. The PCR product was subjected to agarose gel electrophoresis, and the amplified fragment was detected. The fragments were gel-recovered and sequenced.

#### 2.2.9. Genome Comparative Analysis

In recent years, the comparative analysis of the genes of Rhodococcus has drawn much attention. Comparative genomics was the research method used. Bacterial evolutionary genomics can understand the evolution of phenotypes, such as species differentiation, habitat adaptation, virulence evolution, and drug resistance development at the genome-level process, as well as understand the growth of different bacteria in different environments and the intrinsic causes of differences. The evolutionary relationship between the Rhodococcus DSSKP-R-001 and other Rhodococcus strains was compared using collinearity, structural variation, and Venn diagrams to visualize the differences between them, including genome alignment using Lastz (version 1.02.00) software. The use of chainNet and other software packages will provide more fragmented alignment results into longer alignments (such as chain, net, and other forms). According to the new alignment blocks, the alignment and relative orientation are arranged in order, and the alignment blocks belonging to translocation, inversion, and trans + inver were found. The relationship between the blocks is determined by comparing the interblock SV regions. Homologous genes that exist in all samples are called the “core gene,” where the common gene is removed, and the other genes are called “dispensable genes.” The “specific gene” is a gene that is specific only in a certain sample of the gene. All nonshared genes and shared genes were combined as the pan gene. Among them, the core gene and specific gene are likely to correspond to the commonalities and characteristics of the samples, which can be used as the basis for the study of functional differences among the samples.

## 3. Results and Discussion

### 3.1. Screening, Identification, and Preservation of Strains

**Figure 1:**
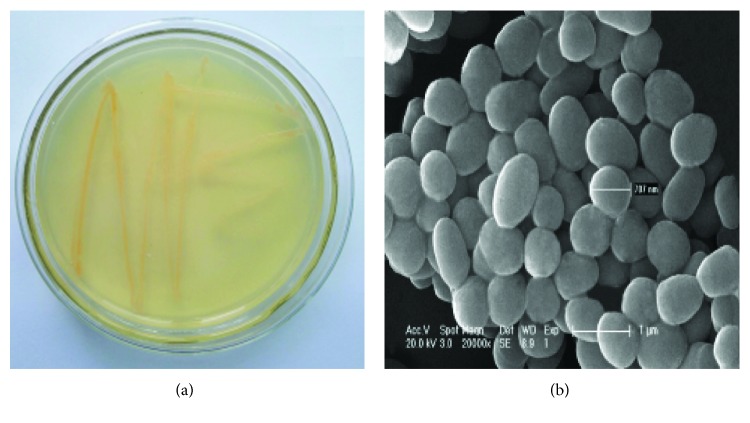
Rhodococcus DSSKP-R-001: (a) growth and (b) electron microscopy.

**Figure 2:**
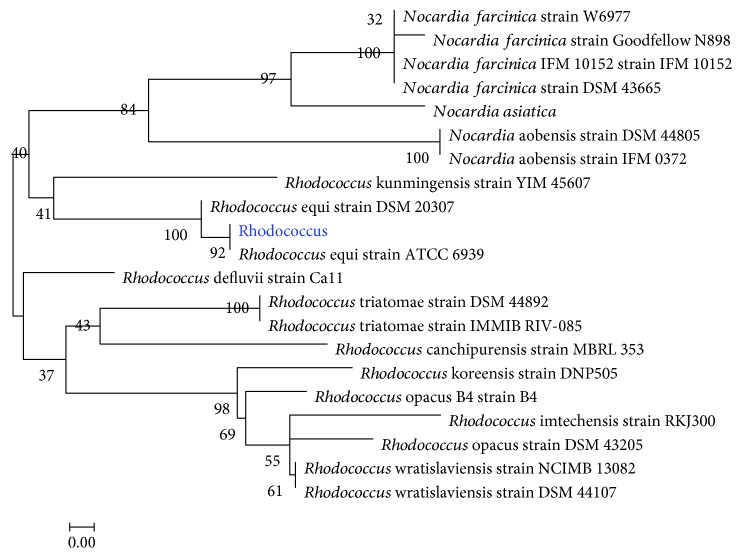
Evolutionary tree based on the 16S rDNA sequence.

A strain of estradiol-degrading bacteria was isolated from the soil near a certain pharmaceutical factory in Beijing. The morphology of the strain was round, slightly convex, smooth, red, and Gram-positive. The strains were spherical and flagellated, as shown in Figure 1. Isolation and identification showed that the estradiol-degrading bacteria in GenBank were compared with the 16S rDNA sequences in GenBank by the BLAST functional module. The 16S rDNA sequences of 20 bacterial species with higher BLAST results were selected, and phylogenetic tree analysis was performed based on Neighbor-Joining using MEGA 5.2.1 software. It can be seen from Figure 2 that the strain is compatible with Rhodococcus equi. The genetic distance is the closest, and it can be preliminarily determined that the strain belongs to Rhodococcus. Rhodococcus DSSKP-R-001 was deposited at the Institute of Genome Microbiology, Accession No. CGMCC No. 12392.

### 3.2. Estradiol Degradation Ability

**Figure 3:**
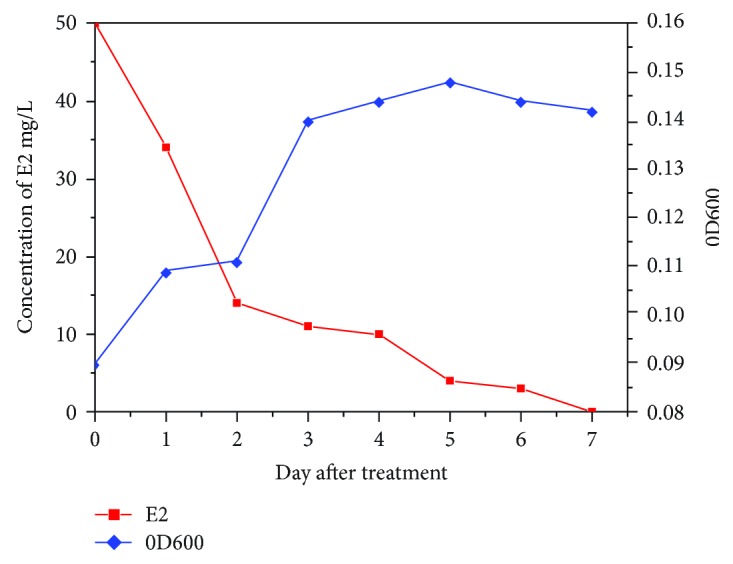
Growth curve and degradation curve of estradiol as the sole carbon source Rhodococcus sp. DSSKP-R-001: 50 mg/L E2, day 3, high-performance liquid phase.

Isolation of the strain Rhodococcus DSSKP-R-001 was done in 50 mg/L estradiol as the sole carbon source of inorganic salt medium, at 30°C at 1200 rpm for 7 days, using the microplate reader to measure the growth of strains, and HPLC to determine the remaining amount of estradiol. The growth curve and degradation curve are shown in Figure 3, and it was observed that we can find strains that can grow well and use of estradiol. On the fifth day, OD600 reached 0.155, with the highest degradation rate of 97% on the third day. From the above results, we can determine that Rhodococcus DSSKP-R-001 can effectively utilize the degradation of estradiol to meet its growth and metabolism needs.

### 3.3. Genomic Characteristics of Rhodococcus DSSKP-R-001

**Figure 4:**
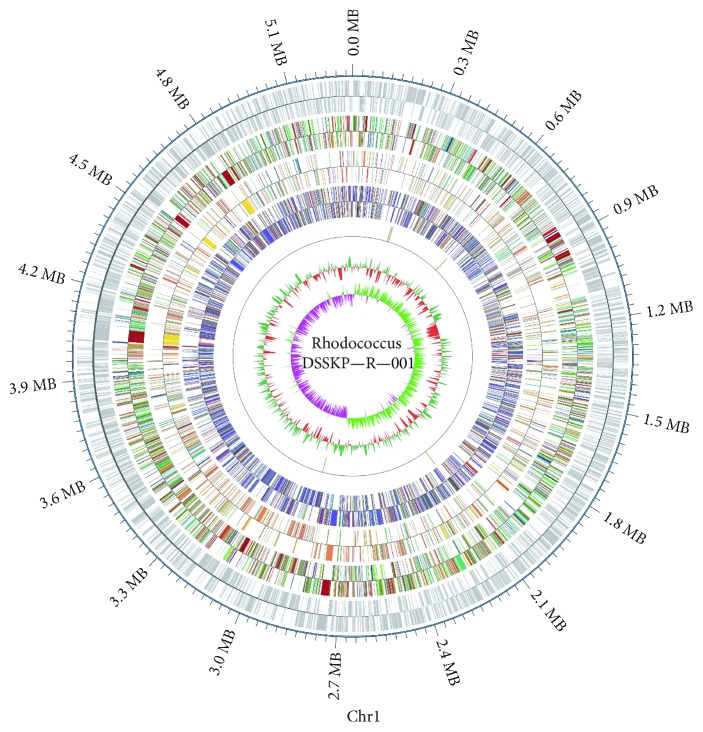
Whole genome map of Rhodococcus strain Rhodococcus sp. DSSKP-R-001.

As shown in Table 2, as a result of genome-wide sequencing and assembly splicing, the genome of the strain Rhodococcus DSSKP-R-001 consists of a circularized chromosome and two circular plasmids. The length of the chromosome was approximately 5.2 M, the size of the plasmid was 0.09 M, the number of G + C was 13, the number of prephage was 6, and the number of CRISPR (Clustered Regularly Interspaced Short Palindromic Repeat Sequences) was 8. The predicted gene and each functional database were BLAST analyzed (Blastp, e − value ≤ 1*E* − 5) using BLAST Results Filtering. For the BLAST results for each sequence, we selected the highest alignment score (default > 40%, coverage = 40%) for the comments. The annotation results included 3736 genes predicted by COG, 2590 genes predicted by KEGG, 3472 genes predicted by GO, 1992 genes predicted by SwissProt, and 4292 genes predicted by NR, and the genome of Rhodococcus was also isolated. The genome sketch of DSSKP-R-001 is shown in Figure 4. The genome is not large, but contains a very rich functional area, the proportion of over 90% of the genome size. In addition to the gene-coding regions, more noncoding regions have functions of transcriptional regulation, posttranscriptional regulation, translational regulation, and epigenetic regulation. Some functional regions are also related to the diversity of species evolution.

The most out-of-band is the genome sequence position coordinates, from outside to inside, respectively. The coding gene, gene function annotation results (according to the actual project situation, may contain COG, KOG, eggNOG, KEGG, and GO database comment results information), ncRNA, genome GC content, and genomic GC skew value distribution.

### 3.4. Estrogen-Degrading Enzyme Gene Analysis

**Figure 5:**
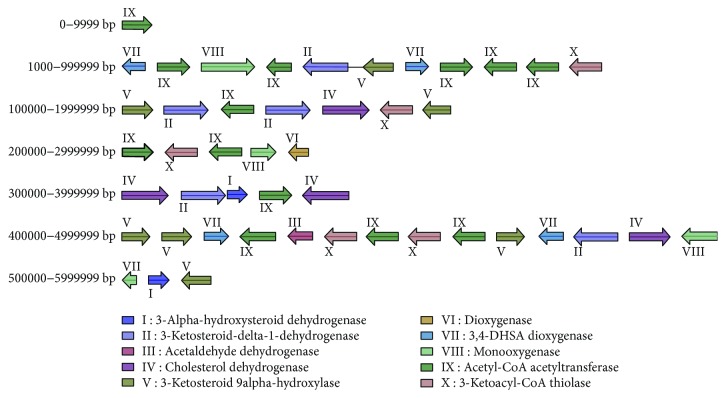
Distribution of estradiol-degrading enzymes in Rhodococcus DSSKP-R-001.

According to the whole genome sequencing results, the BLAST online analysis was performed to compare the amino acid sequence of the strain Rhodococcus DSSKP-R-001 with the gene annotation results obtained from the COG, GO, KEGG, NR, and Swiss-Prot databases, respectively. The homology of the related genes of the published estrogen-degrading enzymes was compared, and the related genes of the estrogen-degrading enzymes were predicted. The results showed that 10 strains of estrogen degradation-related enzymes were predicted by the strain Rhodococcus DSSKP-R-001, as show in Figure 5. Through functional gene annotation and sequence alignment, the length of 3*α*-hydroxysteroid dehydrogenase is 820 bp, repeated twice, and the length of 3-ketosteroid-1-dehydrogenase is 2230 bp 5 times, the length of acetaldehyde dehydrogenase was 945 bp, which appeared once, the length of the cholesterol oxidase was 2127 bp, which was repeated 4 times, and the length of 3-keto steroid-9*α* hydroxylase was 1594 bp, repeated 5 times. The length of the oxygenase was 768 bp, and it appeared once. The length of 3,4-DHSA dioxygenase was 1319 bp, repeated 4 times. The length of monooxygenase was 1260 bp and appeared repeatedly 4 times. The length of the enzyme acetyl-CoA acetyltransferase was 1215 bp, repeated 13 times. The length of 3-ketoacyl-CoA thiolase was 1194 bp, which was repeated 4 times. We can observe that the estradiol-degrading genes are distributed throughout the genome of Rhodococcus DSSKP-R-001 and the frequency of each enzyme is higher. This is likely the reason that estradiol is highly degradable by Rhodococcus DSSKP-R-001.

### 3.5. Estrogen Degradation Enzyme Gene Cloning

**Figure 6:**
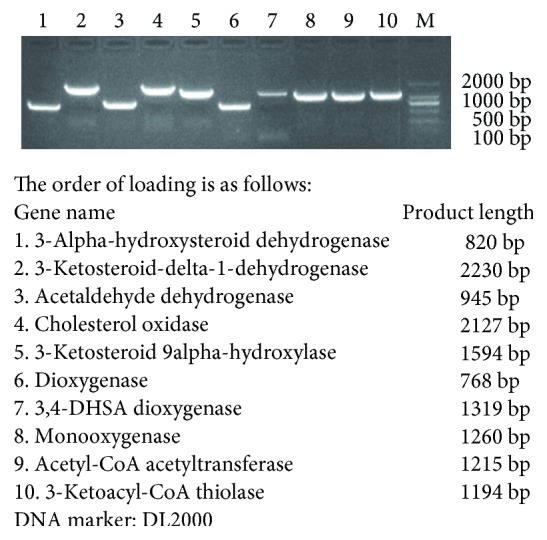
PCR agarose gel electrophoresis.

A total of ten estradiol-degrading enzymes were annotated in Rhodococcus DSSKP-R-001, and ten enzymes were confirmed by PCR. Primers were designed according to the gene sequences obtained by whole genome sequencing. The genes were cloned by PCR and obtained by PCR. The PCR amplification results for the corresponding target fragment are shown in Figure 6. Ten enzyme bands are clearly visible, and the size of each gene fragment is also consistent with the sequence length of the sequencing results. Next, the gel was recovered and sequenced. The sequencing results were compared with the whole genome sequencing results. The results showed that the similarity of all the genes reached more than 90%, and further validation of the whole genome sequencing results is necessary, namely, strain Rhodococcus DSSKP-R-001, in the presence of estradiol degrading enzyme.

### 3.6. Estrogen Degradation Enzyme Gene mRNA Expression

**Figure 7:**
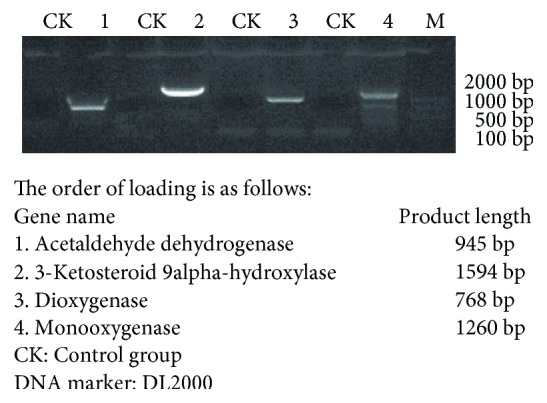
RT-PCR agarose gel electrophoresis.

According to the genome-wide sequencing results, primers were designed and ten esterolytic enzymes of the genes were subjected to RT-PCR. The corresponding target fragments were obtained by RT-PCR. The results of RT-PCR amplification are shown in Figure 7. The results of the comparative PCR showed that the size of four bands corresponded with the sequencing results. Then, the fragments were recovered and sequenced. The results showed that when DSSKP-R-001 was cultured in medium with E2 as a sole carbon source, keto-steroid-9*α*-hydroxylase, monooxygenase, dioxygenase, and aldehyde dehydrogenase were used to investigate the mRNA expression of 3-ketosteroid-9*α* hydroxylase, monooxygenase, dioxygenase, and aldehyde dehydrogenase expression. Of the ten steroid-degrading enzymes present in DSSKP-R-001, four are expressed during induction with estradiol. The three keto-sterol-9 alpha hydroxylated enzymes are repeated five times throughout the sequence, monooxygenase and dioxygenase are repeated four times, and acetaldehyde dehydrogenase appeared once. From the number of enzymes and the occurrence number, we can conclude that DSSKP-R-001 can degrade E2 efficiently. RT-PCR coexpressed four enzymes, six of which were not expressed. These ten enzymes are steroid-degrading enzymes. In this experiment, estradiol is used as a substrate for induction. If induced by other steroid hormones, there may be different kinds of enzyme expression. In the future, we will further investigate the related enzyme genes related to estradiol degradation in DSSKP-R-001 by transcriptome sequencing.

### 3.7. Comparative Genome Analysis

**Figure 8:**
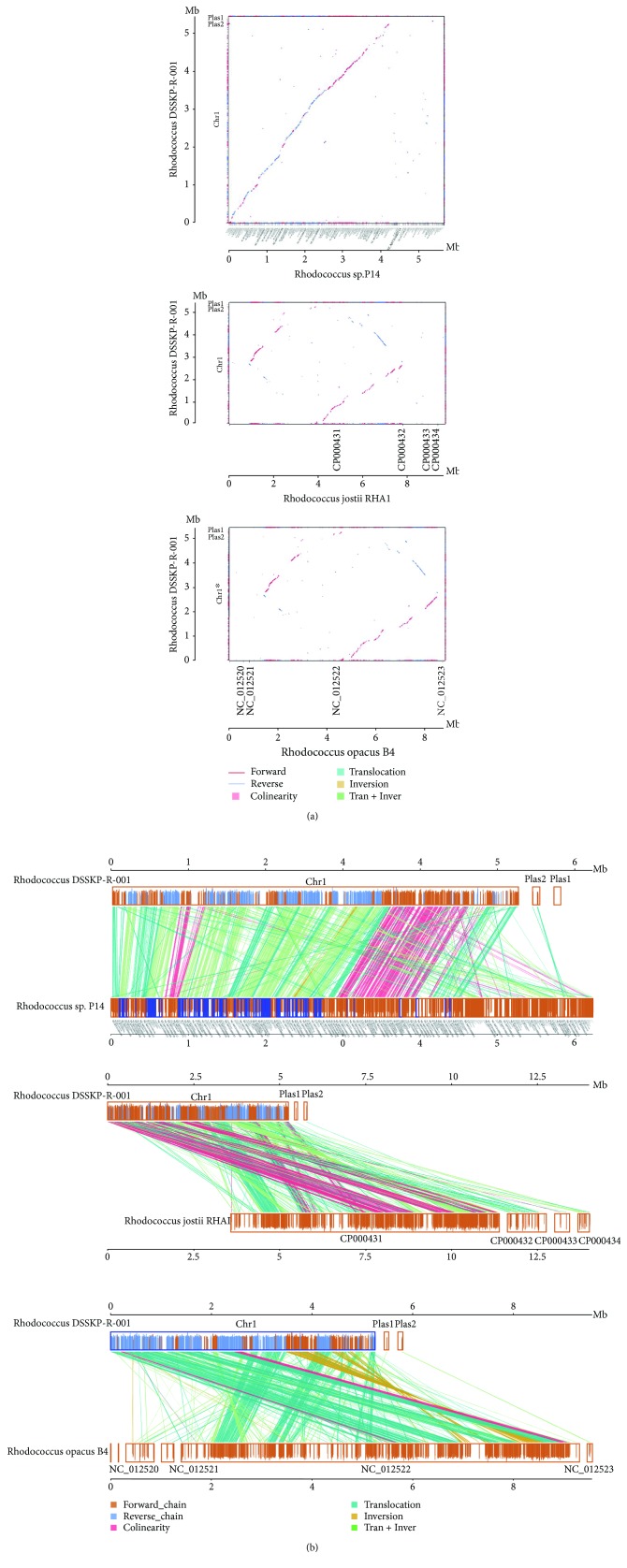
The results of whole genome collinearity. (a) Whole genome two-dimensional collinearity display. The horizontal and vertical axes of the figure correspond to the whole genome (vertical axis sequence name in the upper right corner of “^∗^” means the use of the sequence of negative pairs); lines represent positive alignment, and blue indicates reverse alignment. The middle of the box color matches the type: Collinear: with linear comparison; Translocation: translocation comparison; Inversion: inverted comparison; and Tran + Inver: translocation and inverted comparison. (b) Whole genome parallel collinearity display. The upper axis and lower axes represent the corresponding whole genome. The yellow box on the upper and lower axes shows the genome forward chain, the blue box represents the genomic reverse chain, the height of the filled color in the box indicates the similarity of the alignment, and full filling indicates a similarity of 100%; the link between the upper and lower axes. The color of the alignment type: Collinear: the same linear comparison; Translocation: translocation comparison; Inversion: inverted comparison; and Tran + Inver: translocation and inverted alignment.

Rhodococcus DSSKP-R-001 was shown to be closely related to Rhodococcus sp. P4 by a genome-wide two-dimensional colinear display with other Rhodococcus species in Figure 8(a) (whereas there was a significant association with Rhodococcus jostii RHA1 reduced collinearity, as shown in Figures 8(B), 8(C), and 8(b)). Genome-wide parallel collinearity shows the same result. The collinear region was defined as the region of homologous genes, which indicates that Rhodococcus DSSKP-R-001 constitutes a mosaic gene that may be shared with other Rhodococcus species. In fact, a closely related strain that appears collinearly is a strong signal for conserved gene function. Therefore, it is highly probable that Rhodococcus strain DSSKP-R-001 has a catabolism function similar to that of Rhodococcus sp. P4, which was reported in 2013 by Rhodococcus sp. P4 to efficiently degrade estradiol. In this study, degradation genes were identified, and knockout mutants were also verified.

**Figure 9:**
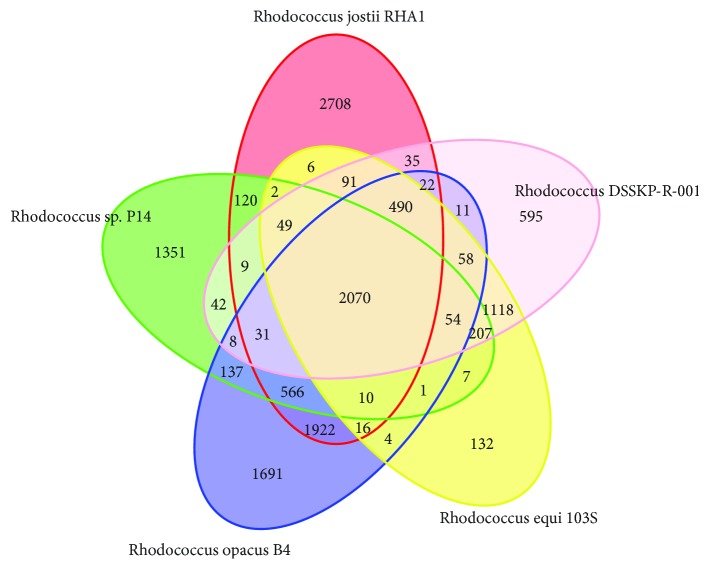
Number of Core/Pan genes in the genome Venn chart. Description: each ellipse represents a sample, the data on each area represents the number of groups that appear only in the sample in this area, and each group represents a group with a greater than 50% similarity, with a sequence length difference of less than 0.7 gene set.

Figure 9 shows the comparison of Rhodococcus DSSKP-R-001 with Rhodococcus sp. P4, Rhodococcus jostii RHA1, Rhodococcus opacus B4, and Rhodococcus equi 103S with a Venn diagram, and it is found that there are 2263, 2797, 2736, and 4137 consensus genes, respectively. There were 2070 homologous genes in the five strains, which also revealed that Rhodococcus was not found in 595 genes of DSSKP-R-001. All nonshared genes and shared genes were combined as a pan gene set of 13,563. Through genome-wide comparative analysis, we found that there is a certain difference and homogeneity among Rhodococcus. When we further study and analyze the homologous genes of these five strains, we found that there are approximately 1159 shared metabolic genes, which also proves that Rhodococcus is a powerful metabolic capacity.

## 4. Conclusion

This article uses interdisciplinary approaches that include comparative genomics, bioinformatics, and metabolism to provide a generalized understanding of the degradation of estradiol-metabolizing genes by Rhodococcus DSSKP-R-001 isolated from the soil. The genome-wide sequencing revealed that the degradative enzymes were widely distributed throughout the genome, and we validated the expression of the enzymes by RTPCR, demonstrating that 3-keto-steroid-9*α* hydroxylase, monooxygenase, dioxygenase, and DSSKP-R-001 are highly expressed in estradiol degradation, which is clearly the reason for the efficient degradation of estradiol by Rhodococcus DSSKP-R-001. Screening of bacterial genomes with sequences of some other Rhodococcus strains by comparative genomics analysis explained a high collinearity with Rhodococcus sp. P4, while an in-depth comparison was made with Rhodococcus sp. P4, Rhodococcus jostii rHA1, Rhodococcus opacus B4, and Rhodococcus equi 103S and 2070 consensus genes were found, indicating that Rhodococcus DSSKP-R-001 constitutes a mosaic gene with other Rhodococcus and shows the function of some conserved genes. This article did not perform gene knockout validation, but future constructions of estrogen degradation engineering bacteria will provide a theoretical basis.

## Figures and Tables

**Table 1:** PCR amplification primers.

Primer name	Sequence (5′ to 3′)	Primer name	Sequence (5′ to 3′)
3a-OH-S	AGCAAGTGACAAAGCCAAACG	3a-OH-AS	GCCATCTTTTCCTACTGTCCCA
3K1D-S	TCAGTCCAGATGTCCCCGATTATG	3K1D-AS	ACGACCACTACACCTTCGCCCT
AD-S	ATGAGCAAGGTCAAGGTCGCG	AD-AS	TCATCGGGTGGCTTCCTTCTTCT
CHO-S	GGGTTCCCGAATCTGTTCCTCA	CHO-AS	GTAACCATCACGCTCCACACGG
3K9A-S	TCTCTGCGGTTCCGTATTTTTC	3K9A-AS	ACGAATCCGTGAGCAGAGCATC
Dio-S	ATGCCGGCCATCTTCCTCAG	Dio-AS	TCAGACCACCTGGAAGGAACGCT
DHSA-S	GTCAGCGGGTGTTGTTGATCTG	DHSA-AS	GCCTTGGTGGGACAGAACAGCA
MON-S	CGGTCCATCAAGGTCGAGCAAC	MON-AS	ATCAGTCAGGTCGTGCTGAGCCT
CoA-S	GTGACCACAGAGGCATTCATCTATG	CoA-AS	TCAGACGCGCTCGATGATG
3-CoA-S	ATGGCCGAGGCAGTCAT	3-CoA-AS	TCAAAGTCGTTCGATGATGG

**Table 2:** Rhodococcus sp. DSSKP-R-001 genomic characteristics.

Characteristic	Genome	Characteristic	Genome
Genome size (bp)	5,438,826	Contig number	3
GC content (%)	68.72%	Genomic islands	13
Chromosome	5,251,559	Contig N50 (bp)	5,252,360
Plas1	92,135	Coding gene assigned to Swiss-Prot	1932
Plas2	95,132	Coding gene assigned to COGs	3736
Gene number	5180	Coding gene assigned to GO	3472
Gene total size (bp)	4,917,591	Coding gene assigned to KEGG	2590

## Data Availability

The data used to support the findings of this study are included within the article.
